# A lightweight model and corn leaf disease recognition

**DOI:** 10.1371/journal.pone.0336945

**Published:** 2025-11-17

**Authors:** Lujie Bai, Shaoqiu Zhu, Haitao Gao

**Affiliations:** 1 College of Information and Network Engineering, Anhui Science and Technology University, Bengbu, China; 2 Research Center of Intelligent Planting and Processing Technology of Crops in Anhui Province, Fengyang, China; Instituto Politecnico Nacional, MEXICO

## Abstract

Corn is a critical food crop globally, widely cultivated due to its strong adaptability. However, it is susceptible to various diseases, necessitating advanced intelligent detection methods to enhance disease prevention, control efficacy, and production efficiency. Traditional disease recognition models suffer from high computational costs or inadequate feature extraction capabilities, making it challenging to achieve efficient and accurate disease identification in complex environments. To improve the accuracy and efficiency of corn leaf disease identification and to meet the requirements of portable devices, this paper proposes a novel ES-ShuffleNetV2 (Exponential Linear Unit + Spatial Group-wise Squeeze-and-Excitation Block) lightweight recognition model for corn diseases. The proposed model builds upon the ShuffleNetV2 architecture. Firstly, an improved attention mechanism, SGSE, is incorporated immediately following the first convolutional layer to emphasize fine-grained features in corn leaf disease images, enhancing the model’s focus on key characteristics. Secondly, the model replaces the ReLU activation function in the down-sampling and basic units with the ELU function, facilitating smoother gradient propagation and faster convergence by allowing a small negative gradient inflow. Additionally, layer pruning techniques are employed to eliminate redundant parameters, reduce model complexity, and enhance operational efficiency on mobile devices. Experimental results demonstrated that the ES-ShuffleNetV2 model achieved recognition accuracy of 97.07%, surpassing the base model’s accuracy of 95.43%. After pruning, the new model reduced parameters by 30.45% and FLOPs by 30.26% compared to the original model, meeting the criteria for a lightweight recognition model. Furthermore, the ES-ShuffleNetV2 model outperformed competing models in Accuracy and F1-Score, validating its effectiveness in corn leaf disease recognition and providing valuable insights for future research.

## 1 Introduction

Corn is one of the most important staple crops globally, widely cultivated around the world for use in feed, industrial materials, and other fields, and holds significant economic value [1]. However, corn production faces threats from various diseases, such as corn leaf spot, Fusarium head blight, and rice blast, among others. The occurrence of these diseases can hinder the expected growth of corn, leading to severe yield reduction or even plant death, posing significant challenges to agricultural production and food security [2]. In traditional agricultural production, disease recognition mainly relies on farmers’ experience and visual observation. However, due to the lack of systematic scientific methods, this approach suffers from significant issues of inaccuracy and inefficiency. Particularly for diseases with early or subtle symptoms or those in complex environments, traditional methods tend to overlook key symptoms, leading to missed optimal control opportunities, thus exacerbating the spread and proliferation of the disease [3].

The adoption of advanced intelligent technologies and tools has become increasingly necessary to improve the precision and efficiency of disease detection. Since the 1980s, machine learning technologies have gradually emerged and been explored for applications in agriculture [4]. Early studies primarily relied on image scanning and traditional image processing techniques to analyze crop diseases caused by environmental stress, such as harmful gas pollution. These studies focused on disease recognition in leaves and stems, as well as monitoring crop growth conditions, demonstrating the feasibility of image analysis methods for crop health management. However, as traditional image processing techniques were increasingly applied to crop disease identification, their limitations in feature extraction capability and recognition accuracy became evident. Background interference in complex environments, the diversity of crop leaf morphologies, and subtle disease symptom changes have made it difficult for traditional methods to meet precise recognition requirements. To overcome these technical bottlenecks, Hinton et al. [5] proposed Deep Belief Networks (DBN) in 2006, marking the formal introduction of deep learning concepts. With its powerful autonomous feature extraction and generalization capabilities, deep learning has achieved remarkable success in areas such as visual perception, audio analysis, and natural language processing, laying a theoretical and technical foundation for innovations in disease recognition technologies.

Recently, deep learning–based approaches for crop disease identification have been widely explored and implemented. Wu Yehui et al. [6] proposed an image recognition method using a lightweight model based on a randomly augmented Swin-Tiny Transformer. By optimizing the Swin-Tiny Transformer model and fine-tuning parameters on a corn disease dataset, an identification accuracy of 93.59% was achieved, with a parameter size of 28.80M. Luo Yang et al. [7] introduced a crop leaf grading and disease recognition algorithm using backbone information sharing and multi-receptive field feature adaptive fusion. Experimental results demonstrated that CLGDRNet achieved recognition accuracies of mAP@0.5 and mAP@0.5:0.95 at 85.0% and 76.1%, respectively, on the early-cured tobacco leaf grading dataset and 97.6% and 74.2%, respectively on the apple leaf disease dataset, with a model size of 5.0M. Liang Xiuman et al. [8] improved the Fire module of the SqueezeNet network by incorporating spatial attention mechanisms and dense connection modules in deeper network layers to improve the ability to extract and reutilize features. Two lightweight CNN models were constructed for identifying diseased apple leaves, achieving recognition accuracies of 89.60% and 94.37%, corresponding to increases of 2.98 and 7.75 percentage points over the original network, with parameter sizes of 0.9M and 2.5M. Yang et al. [9] developed a self-supervised multi-network fusion classification model to identify common strawberry disease types, attaining an accuracy of 92.48%. Ahmad Loti Nurul Nabilah et al. [10] utilized deep learning–based feature extraction techniques to detect key disease and pest traits from chili leaf images. The features were fed into machine learning classifiers, achieving a best accuracy of 92.10%. Azgomi Hossein et al. [11] employed a multilayer perceptron neural network for apple disease recognition, achieving a best accuracy of 73.7%. Jixia H et al. [12] employed remote sensing datasets of pine wood nematode disease to train five well-known models—AlexNet, GoogLeNet, SqueezeNet, ResNet-18, and VGG16—using transfer learning. A combined macro-architecture and micro-module adjustment strategy was used to improve the model structure. Experimental findings indicated that the enhanced SqueezeNet model delivered the highest recognition efficiency and accuracy, achieving 94.90%. These studies emphasize that crop disease recognition accuracy requires further enhancement to satisfy the needs of practical applications. Chen Yu et al. [13] proposed the YOLOv5-CBM model for tea leaf disease recognition, achieving an accuracy of 97.3% with a parameter size of 26.8M. Huang Lüwen et al. [14] designed a tea leaf disease recognition model, CBAM-TealeafNet, based on discrete wavelet transform (DWT) and MobileNetV3 fusion, achieving an overall recognition accuracy of 98.70% for five distinct tea leaf diseases, having a parameter size of 3.16M. Sun Wenbin et al. [15] introduced a disease recognition approach utilizing visible spectra combined with an enhanced attention mechanism, designing a new attention module (SMLP) and a crop disease recognition model (SMLP_ResNet). The disease recognition rates on the two datasets reached 86.93% and 99.32%, respectively, with a model weight size of 48.6MB. Shweta Bondre et al. [16] proposed the IFMR-CNN method, achieving a 96% accuracy in disease localization and classification. Madakannu Arun et al. [17] developed an improved LeNet architecture based on deep convolutional neural networks (CNN) for classifying corn leaf diseases, achieving an accuracy of 97.89% after multiple experiments. Karlekar et al. [18] proposed a soybean leaf disease recognition method for complex backgrounds employing deep learning techniques. They designed a visual data processing module (IPM) to remove complex backgrounds and segment leaf areas before CNN network training, attaining a recognition accuracy of 98.14%. Jing Jiaping et al. [19] introduced the BC-YOLOv5 method in the recognition of tomato diseases, achieving 95% accuracy. Lei Tang et al. [20] introduced an enhanced multi-scale inverse bottleneck residual network, based on ResNet-50 and incorporating a triplet parallel attention mechanism, achieving 98.73% accuracy on the apple leaf disease dataset with 116.3M parameters. Jinsheng Deng et al. [21] presented the Ghost ResNeSt-Attention RReLU-Swish network model (GR-ARNet) for banana leaf disease recognition. They introduced a novel K-level VisuShrink algorithm (KVA) for denoising banana leaf images, achieving an average accuracy of 96.98%. Despite achieving high accuracy, these models often have large parameter sizes, necessitating further optimization to reduce model size, shorten inference time, and further enhance recognition accuracy.

In summary, applying deep learning-based object detection techniques for corn disease identification is feasible; however, specific challenges still require attention.

1. Traditional neural network models, such as ResNet and vision transformers (e.g., Swin Transformer [22]), exhibit strong feature extraction capabilities but are limited by high complexity, characterized by large parameter sizes and significant computational costs. These constraints impede their deployment on mobile devices. Consequently, lightweight models play a critical role in reducing inference time and enhancing recognition speed, thus fulfilling the requirements for efficient, lightweight, and real-time applications in agricultural settings.
2. Lightweight models such as ShuffleNet, SqueezeNet, and the MobileNet series effectively reduce computational costs but face limitations in feature representation. These models often struggle to capture global features of disease data, leading to lower recognition accuracy and a tendency to overlook detailed information during detection. Additionally, complex background interference in real-world data poses further challenges to accurate disease identification.

This paper proposes a lightweight disease recognition model, ES-ShuffleNetV2, to address the shortcomings of current methods. The main contributions of this work are as follows:

1. Based on ShuffleNetV2, an improved SGSE attention mechanism is introduced after the first convolution layer, focusing on fine-grained features in corn disease samples. This enhancement enhances the model’s capability to represent features and improves disease recognition accuracy in complex backgrounds.
2. Employing the ELU activation function in both the basic and down-sampling units enhances the model’s nonlinear expression capability, thereby accelerating network convergence and improving disease recognition performance.
3. A layer pruning strategy is introduced to effectively reduce redundant parameters, decrease model complexity, and enable efficient inference.

Testing on a dataset containing six types of corn diseases demonstrates that the proposed model markedly enhances accuracy and efficiency of corn leaf disease recognition while maintaining its lightweight design. These results provide a novel technical approach for the development of innovative precision agriculture.

## 2 Dataset collection and construction

### 2.1 Dataset image collection

Common corn diseases comprise extensive spot diseas, small spot disease, rust, gray leaf spot, northern corn leaf blight, stalk rot, and corn smut, among others [23]. This study focuses on six typical corn diseases—Common rust, Bipolaris maydis, Curvularia lunata(wakker) boed spot, Northern leaf blight, Own spot, and Sheath blight—as the subjects for disease recognition analysis [24–26].

The practical dataset used in this study was sourced from the Anhui Academy of Agricultural Sciences, with data collection conducted in corn fields located in the northern region of Anhui Province. To ensure the authenticity and representativeness of the data, the research team utilized camera equipment to capture images of diseased corn plants from multiple angles. The shooting environment was complex, with the background including factors such as straw, soil, and weeds, simulating the actual field conditions. The camera model used was the NIKON D810, with images captured at a resolution of 1001x1500 pixels.

**Fig 1:**
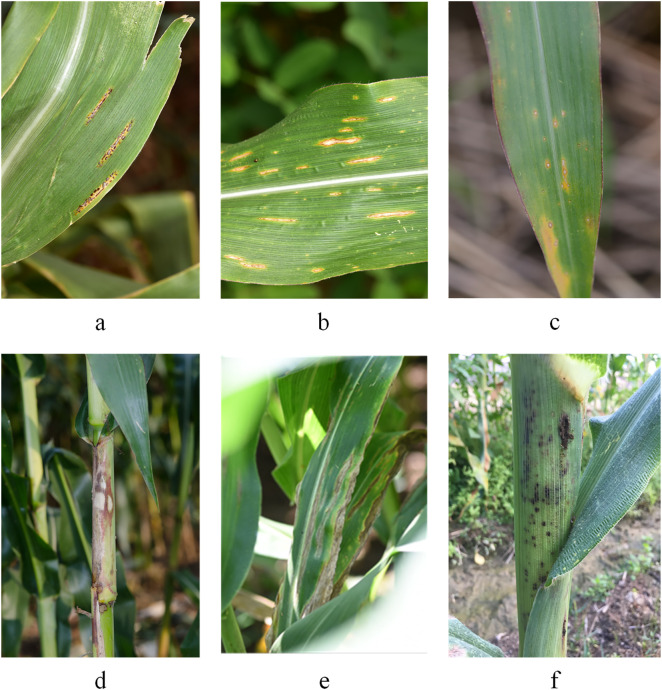
Sample of corn disease. a: Common rust,b: Bipolaris maydis,c: Curvularia lunata(wakker) boed spot,d: Northern leaf blight,e: Own spot,f: Sheath blight.

The dataset comprises a total of 2,725 images of different corn leaf diseases, including 470 of Common rust, 645 of Bipolaris maydis, 260 of Curvularia lunata (Wakker) leaf spot, 546 of Northern leaf blight, 356 of Own spot, and 448 of Sheath blight. Sample images from the dataset are presented in Fig 1.

### 2.2 Dataset construction

**Fig 2:**
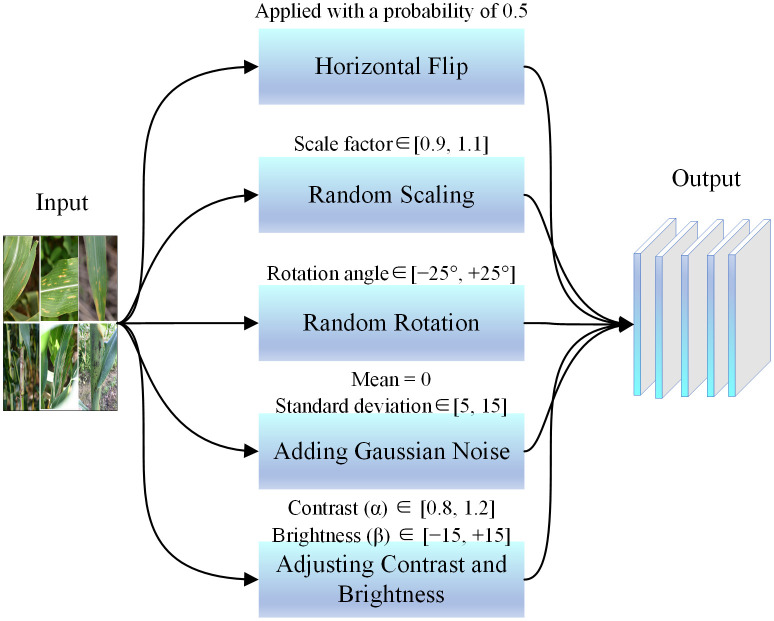
Dataset augmentation.

The collected raw images underwent data augmentation, as shown in Fig 2, which included operations such as horizontal flipping, random scaling, random rotation, adding Gaussian noise, and contrast adjustment. The specific parameter ranges employed for data augmentation techniques, such as rotation, scaling, and noise addition, are clearly presented in Fig 2. These operations enhance the robustness of the samples to a certain extent, improving the model’s generalization ability for corn leaf disease recognition under natural conditions, while also boosting the target detection algorithm’s ability to remember labels. To decrease the need for computational resources and speed up training, the images were resized to 256 × 256 pixel resolution. Through data augmentation and sample normalization, a total of 10,062 corn disease images were obtained.

**Fig 3:**
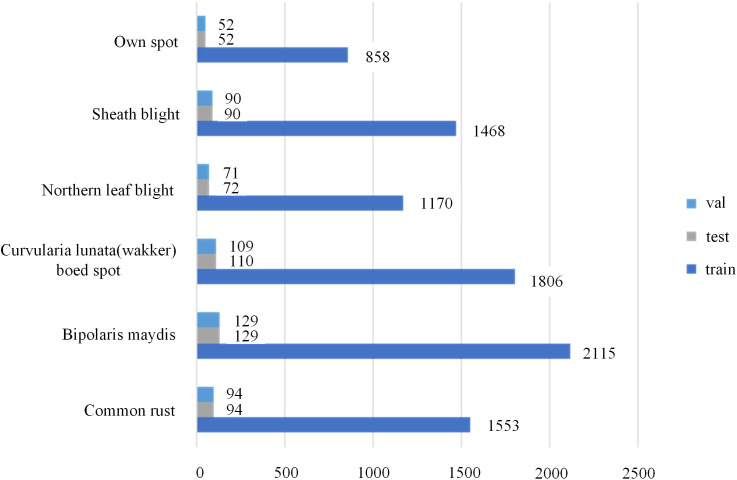
Sampling of major corn disease samples.

To prevent data leakage, the original dataset was randomly divided into training, validation, and test subsets in a 6:2:2 ratio. Data augmentation was applied exclusively to the training set, while the validation and test sets were subjected only to normalization. The detailed distribution of images across each subset is illustrated in Fig 3. This procedure ensured the proper construction of the experimental dataset.

## 3 Construction of the ES-ShuffleNetV2 detection model

**Fig 4:**
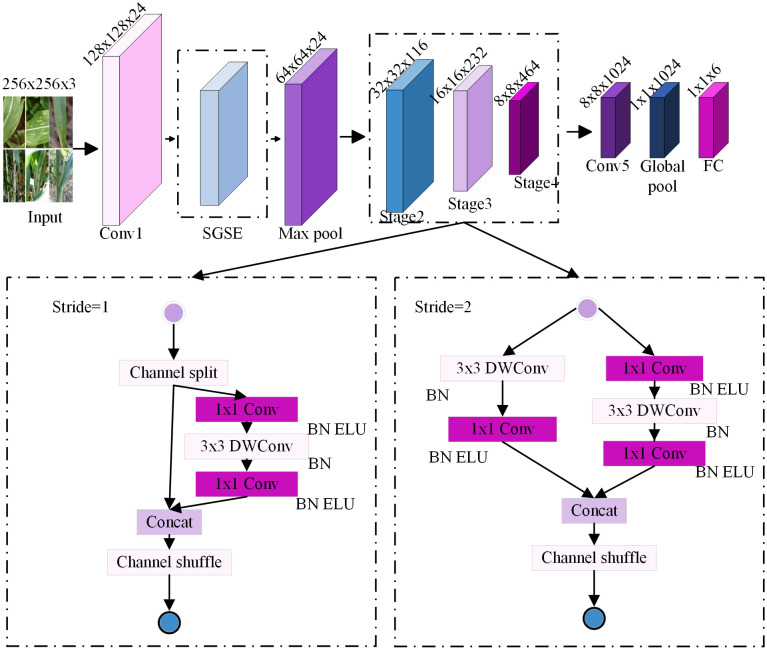
Framework structure of ES-ShuffleNetV2 network model.

The ES-ShuffleNetV2 model improves upon ShuffleNetV2. It integrates the ELU activation function and the enhanced SGSE attention mechanism while employing model-pruning techniques to eliminate redundant parameters. This approach addresses the challenges of high resource consumption, insufficient feature selection, and low accuracy in traditional deep-learning models for corn leaf disease recognition. The model enhances recognition accuracy while maintaining computational efficiency, making it suitable for resource-constrained corn leaf disease recognition scenarios. The ES-ShuffleNetV2 model is illustrated in Fig 4.

### 3.1 ShuffleNetV2 base model

In the task of corn leaf disease recognition, the ShuffleNetV2 model serves as an efficient, lightweight convolutional neural network, providing an effective solution for real-time image analysis in environments with limited resources, such as mobile devices and embedded platforms. ShuffleNetV2 was proposed by Zhang et al. in 2018 [27] as an optimized version of the original ShuffleNet, with its network architecture shown in Table 1. The model significantly reduces computational complexity and memory access requirements while maintaining strong feature representation capabilities through mechanisms such as group convolution, depthwise separable convolution, channel split, and channel shuffle.

**Table 1:** Architecture of the ShuffleNetV2 network.

Layer	Output size	Kernel size	Stride	Repetitions	Output channels
Conv+BN + ReLU	112x112x24	3x3	2	1	24
Max Pooling	56x56x24	3x3	2	1	24
Stage2	56x56x116	3x3	2	3	116
Stage3	28x28x232	3x3	2	7	232
Stage4	14x14x464	3x3	2	3	464
Conv+BN + ReLU	7x7x1024	1x1	1	1	1024
Global Average Pooling	1x1x1024	–	–	1	1024
Fully Connected+Softmax	1x1xN	–	–	1	N

Specifically, ShuffleNetV2 initially splits the input feature channels into two portions: a portion extracts fine-grained disease features through depthwise separable convolutions. In contrast, the remaining portion preserves the continuity of the information flow via skip connections. Subsequently, the channel shuffle mechanism recombines the two parts of the features, facilitating information exchange across different feature dimensions and enhancing both feature diversity and inter-channel relationships. This design not only effectively reduces information redundancy but also significantly improves the model’s performance in identifying corn disease features. Furthermore, the model’s lightweight bottleneck module minimizes redundant calculations in group convolutions, lowering the parameter count and enhancing suitability for memory-limited settings. Therefore, this paper selects it as the base model.

### 3.2 Feature extraction enhancement module

**Fig 5:**
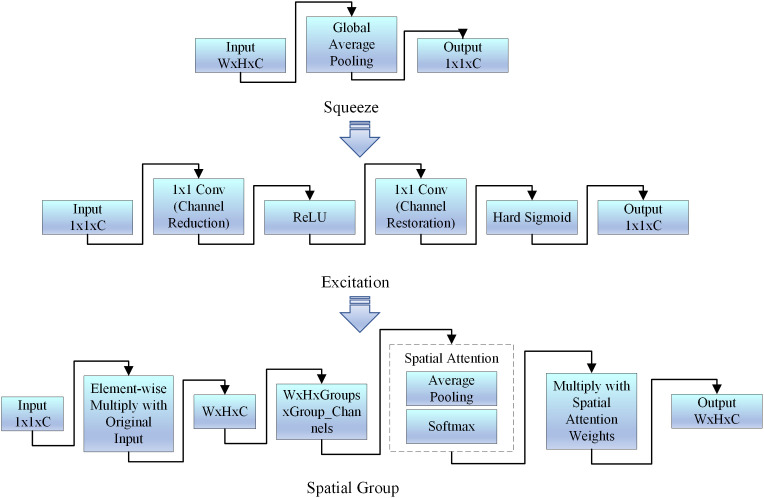
The SGSE module.

Currently, attention mechanisms such as spatial, channel, and convolutional attention have been widely applied to enhance feature extraction [28]. However, these methods frequently result in higher model complexity and lower operational efficiency. To strike a balance between recognition accuracy and real-time efficiency, this study presents a novel lightweight feature enhancement module called the SGSE module, as shown in Fig 5. This module combines the properties of Squeeze-and-Excitation (SE) and Spatial Group-wise Enhancement (SGE) mechanisms. By leveraging the synergistic effects of channel and spatial attention, it enhances feature extraction while minimizing interference, all without adding extra computational burden. The SE module initially gathers global features for each channel through global average pooling, then produces channel attention weights with two 1 × 1 convolution layers, effectively emphasizing the input features. To enhance the depiction of local features, the SGE module partitions the feature map into multiple groups and calculates spatial attention for each. Using the Softmax function, the attention weights are normalized, enabling spatial dimension feature enhancement. Additionally, a new Hard-Sigmoid function is introduced in the attention weight calculation process. Compared to the traditional Sigmoid function, Hard-Sigmoid [29] offers a nearly linear response and higher computational efficiency, effectively reducing the model’s computational overhead while maintaining a nonlinear mapping of the weights. Ultimately, the module outputs features that have been jointly enhanced along both channel and spatial dimensions, efficiently capturing significant regions and detailed information within the image. Experimental results demonstrate that the SGSE module significantly improves model performance in corn leaf disease recognition tasks.

#### 3.2.1 SE attention module.

The SE attention module [30,31] adapts the channel weights to help the network focus on important attributes including color, texture, and form, reducing background interference and improving disease classification accuracy.

**Fig 6:**
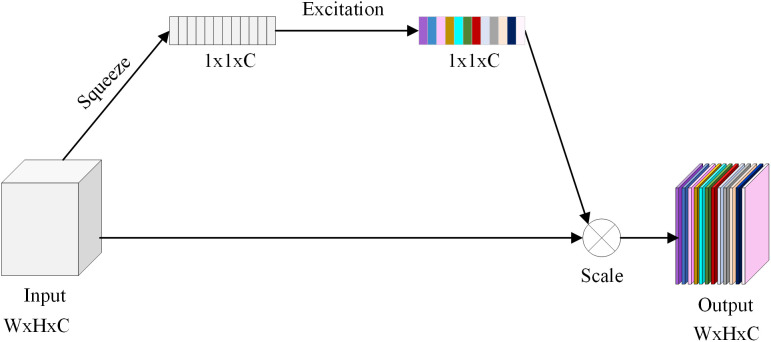
The network structure of SE (Squeeze-and-Excitation).

Fig 6 shows the principle of the SE attention mechanism, which consists of two operations: Squeeze and Excitation.

Squeeze: The global average pooling (GAP) operation is applied to compress the feature map of each channel into a scalar. Specifically, for a given input feature map 𝑋∈𝑅^𝐻^^×𝑊×𝐶^ (where 𝐻 and 𝑊 are the height and width of the feature map, and 𝐶 is the number of channels), the global average pooling operation computes the mean of each channel, resulting in a vector 𝑧 of length 𝐶:


$${𝐳}_{c}=\frac{\text{1}}{H\times W}\sum_{i=1}^{H}\sum_{i=1}^{W}X_{ijc}$$ (1)

Excitation: This operation learns the importance of each channel through a simple, fully connected network, typically a two-layer small MLP. First, the compressed feature vector 𝑧 is input into a fully connected layer with a ReLU activation function. Then, a Sigmoid activation function outputs the weight for each channel. Finally, the learned channel weights 𝑠 are multiplied by the original feature map 𝑋 to achieve channel recalibration:


$$𝐬=\sigma (W_{2}\cdot Re\mathrm{\backslash nolimits}LU(W_{1}\cdot 𝐳))$$ (2)

$$X_{ijc}^{\prime }=X_{ijc}\cdot {𝐬}_{c}$$ (3)

${𝐳}_{c}$ denotes the global average feature of channel c, H denote the feature map’s height, W denote the feature map’s width, c is the channel count, *X* is the input feature map, $W_{\text{1}}$and $W_{\text{2}}$ are the weights of the fully connected layer, σis the Sigmoid activation function, $𝐬\in {𝐑}^{c}$ is the channel weight vector.

In this way, the SE module adaptively assigns different weights to different channels, enhancing critical feature representations while suppressing less informative ones. (The above formulation follows Hu et al., 2018.).

#### 3.2.2 SGE attention mechanism module.

The Spatial Group-wise Enhance (SGE) module enhances the model’s ability to represent image features by performing spatial grouping and local enhancement on the feature map [32].

**Fig 7:**
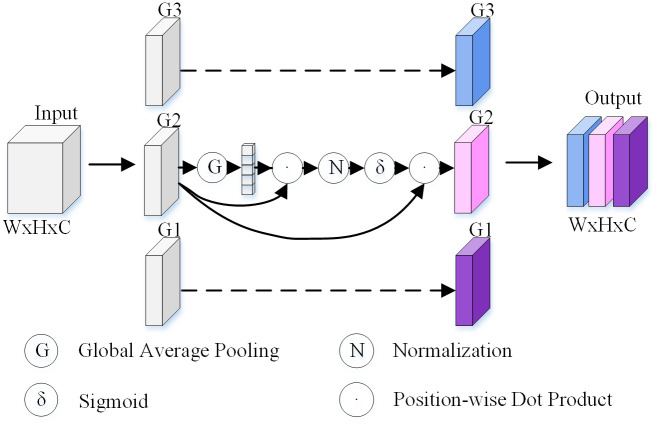
The network structure of SGE (Spatial Group-wise Enhancement).

As illustrated in Fig. 7, the input tensor is divided into 𝐺 groups along the channel dimension, where each group contains 𝐶/𝐺 channels. This grouping helps to reduce computational complexity and introduces spatial attention to each group, allowing the model to capture local information at different positions and fine-tune the extraction of fine-grained features in disease regions.

For each group, average pooling is performed along the channel dimension to obtain the spatial response map, which is then normalized using the softmax function to generate spatial attention weights. The recalibrated group feature map is computed as:


$${\tilde{X}}^{\left(k\right)}=A^{\left(k\right)}⊙X^{\left(k\right)}$$ (4)

where $A^{\left(k\right)}$ denotes the spatial attention weights of the k−th group obtained through softmax normalization, and ⊙ represents element-wise multiplication, ${\tilde{X}}^{\left(k\right)}$ represents the recalibrated feature of the k−th group.

Through this mechanism, SGE amplifies important regional features and suppresses irrelevant ones, thereby enhancing the fine-grained feature extraction capability of the model. (The above formulation follows Li et al., 2019.).

### 3.3 Training performance enhancement function

Traditional fully convolutional neural networks typically use the ReLU activation function. However, in corn leaf disease recognition, the disease features often occupy relatively small regions in the image. The use of ELU activation function can effectively accelerate model convergence and improve noise resistance, thus improving the model’s capacity to capture subtle disease characteristics [33]. Compared to the ReLU function, the smooth transition of the ELU function in the negative region allows for more stable gradient propagation, preventing the issue of zero gradients in the negative region of ReLU. This smooth transition in the negative region, which allows for more stable gradient propagation and prevents the issue of zero gradients in the negative region of ReLU, leads to faster model training, better convergence, and improved model accuracy and generalization ability [34].

**Fig 8:**
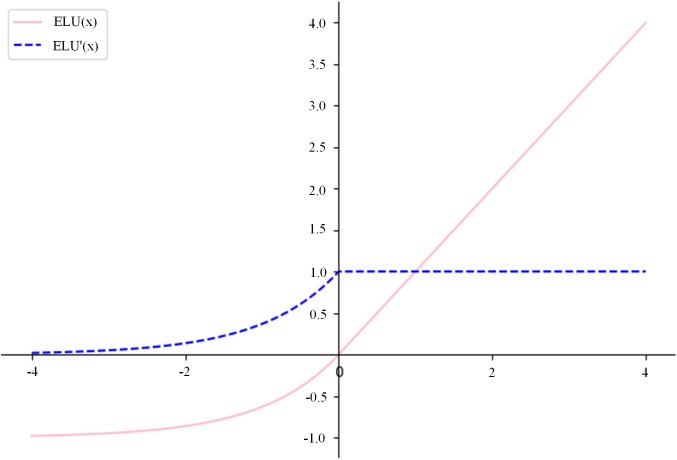
ELU activation function and its derivative.


The graph of the ELU activation function and its derivative is shown in Fig 8. The calculation is defined as:

$$ELU\;(x)=\left\{ \begin{array}{c} x,\;ifx>\text{0}\\ \alpha \left(e^{x}-\text{1}\right),ifx\leq \text{0} \end{array}\right.\;$$ (5)

and its derivative is expressed as:


$$ELU\;(x)=\left\{ \begin{array}{c} \text{1},\;ifx>\text{0}\\ \alpha e^{x},ifx\leq \text{0} \end{array}\right.\;$$ (6)

x is the input, usually a weighted sum of neurons. α is a hyperparameter that controls the slope of the negative part. It is usually set to α=1. (The above formulation follows Clevert et al., 2015.)

### 3.4 Model parameter optimization methods

Due to their large number of parameters, existing models often suffer from high computational and memory demands, which makes efficient deployment on resource-limited mobile devices difficult [35–37]. To address the issue of high computational and memory costs associated with existing models, we employ model layer pruning in this paper to remove redundant parameters. This reduction in model complexity decreases the computational load and memory usage during inference, ultimately achieving lightweight performance for improved efficiency on mobile devices. Additionally, layer pruning reduces overfitting and improves the model’s generalization performance, thus maintaining performance while significantly improving operational efficiency.

**Fig 9:**
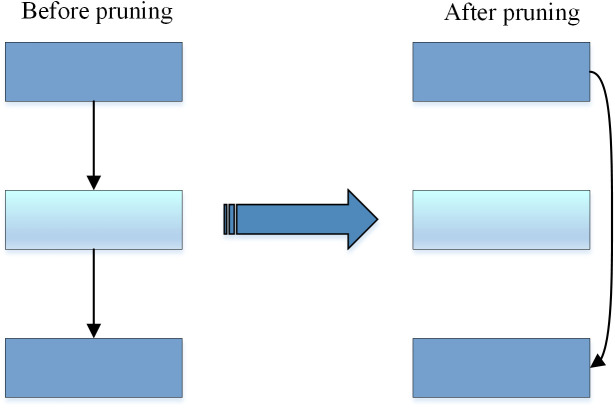
Schematic diagram of layer pruning.

In this paper, the basic ShuffleNetV2 network consists of three stages (see Table 1). The second and fourth stages consist of one down-sampling unit followed by three basic units, whereas the third stage includes one down-sampling unit and seven basic units. Layer pruning is applied to remove redundant intermediate layers, resulting in a more lightweight model, as shown in Fig 9. In the final design, the second and fourth stages each keep one down-sampling unit and one basic unit, while the third stage preserves one down-sampling unit along with two basic units. This modification significantly reduces computational complexity while preserving model performance.

### 3.5 Model recognition flowchart

**Fig 10:**
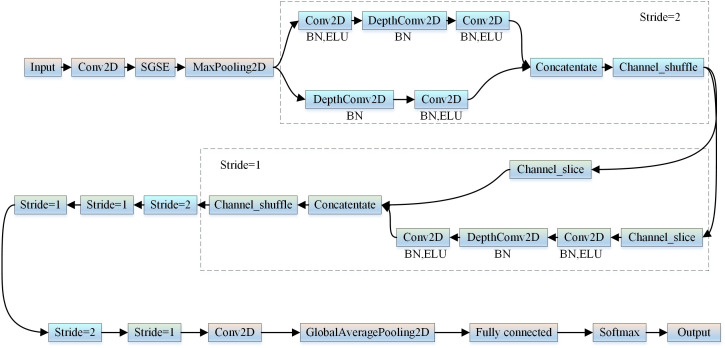
Model recognition flowchart.

The specific implementation steps of the ES-ShuffleNetV2 disease recognition model developed for corn diseases are shown in Fig 10. The corresponding pseudocode of this implementation is summarized in Table 2.

**Table 2:** Pseudocode describing the implementation workflow of the ES-ShuffleNetV2 model.

Algorithm1: Dataset Splitting, Offline Augmentation, and Model Training Procedure of ES-ShuffleNetV2
**Input:** Raw corn disease images organized by class (RawImages)
**Output:** Split dataset (split_raw), augmented training data (augmented_dataset), trained model weights, and evaluation metrics
1: **Begin** 2: Initialize random seed = 42 3: Set train_ratio = 0.6, val_ratio = 0.2, test_ratio = 0.2 4: **FOR** each class c in RawImages DO 5: Split images into train, val, and test subsets using train_test_split() 6: Copy files into split_raw/ {train, val, test}/c 7: **END FOR** 8: Save split information to split.json 9: **FOR** each image x in split_raw/train DO 10: Resize x to 256 × 256 and normalize pixel values 11: Apply random augmentation operations: - horizontal flipping (applied with a probability of 0.5) - random scaling (scale factor randomly sampled from [0.9, 1.1]) - random rotation (rotation angle uniformly sampled from [−25°, + 25°]) - Gaussian noise addition (zero-mean Gaussian noise with standard deviation randomly selected from [5, 15]) - brightness and contrast adjustment (contrast scaling factor α randomly sampled from [0.8, 1.2], brightness offset β randomly sampled from [−15, + 15]) 12: Save augmented images to augmented_dataset/train_aug 13: **END FOR** 14: **FOR** val and test sets: Resize and normalize only (no augmentation) 15: Construct ES-ShuffleNetV2 model: Input → Conv2D → SGSE Module → MaxPooling2D → multiple v2_blocks → Conv2D (1 × 1) → GlobalAveragePooling2D → FullyConnected → Softmax 16: Compile model using SGD (Learning rate = 0.01, momentum = 0.9, decay = 8 × 10^−4^) 17: Train model for 60 epochs with ReduceLROnPlateau and EarlyStopping 18: Evaluate model on test set to compute Accuracy, Precision, Recall, and F1-Score 19: Calculate FLOPs and Parameters, compare with baseline model 20: Save trained weights and evaluation metrics 21: **END**

Step 1: Standardize the size of the input corn disease images to 256x256 pixels, with the input tensor shape of [256, 256, 3], and normalize the pixel values in each channel.

Step 2: Extract preliminary features through a Conv2D layer with a kernel size of 3x3, a stride of 2, and 24 output channels, using the ReLU activation function to introduce non-linear feature expression capabilities.

Step 3: Apply the SGSE module to enhance the feature map with spatial and channel attention. The module uses global average pooling, channel weight computation, weighted operations, and spatial group enhancement to focus on key features. The results are fused with the convolution output to improve feature representation.

Step 4: For spatial down-sampling, use a MaxPooling2D layer with a 3x3 pooling window and a stride of 2, preserving significant features while reducing computational load.

Step 5: In the core feature extraction phase, the model further extracts and enhances features through multiple v2_block modules. In each module, the input feature map is divided into two branches, where depthwise separable convolutions are applied to extract different spatial features. The channels are restored using 1x1 convolutions, and the ELU activation function enhances the model’s non-linear expression. The features from both branches are then concatenated and channel-shuffled to improve inter-channel feature interaction, progressively extracting richer feature representations.

Step 6: After feature extraction, compress the features using a Conv2D layer. When the scale factor is 2, the output channel count is 2048; otherwise, it is 1024. The kernel size is 1x1, with a stride of 1.

Step 7: Use GlobalAveragePooling2D to convert the feature map into a one-dimensional feature vector, extract global semantic features, and map them to the 6 corn disease categories using a fully connected layer. The output is a probability vector of shape [6].

Step 8: Apply the softmax activation function to convert the output into category probabilities, indicating the likelihood of the input image belonging to each category.

Step 9: The model predicts the disease category of the input image based on the probability vector, enabling automatic identification and classification of corn diseases.

## 4 Experimental designs

### 4.1 Experimental platform

The operating system used in this test platform is Windows 10, the CPU is Intel(R) Core (TM) i5-10400F, and the GPU is NVIDIA GeForce GTX 1650 with 16GB memory. The test software environment includes Anaconda IDE, Python 3.6.2, TensorFlow 1.14.0, and CUDA 10.0.

### 4.2 Evaluation metrics

In classification tasks, dataset imbalance is a common issue, where the accuracy comes from categories with many samples, often resulting in incorrect classification of minority class samples [38]. To thoroughly assess the performance of the proposed ES-ShuffleNetV2 model, we use Accuracy, Precision, Recall, and F1-Score as evaluation metrics. Their formulas are expressed as follows:

Accuracy represents the ratio of correctly classified samples to the total number of samples in a classification task.


$$Accuracy=\frac{TP+TN}{TP+TN+FP+FN}$$ (7)

Precision represents the proportion of samples that are correctly predicted by the classification model among those labeled as positive in the test set.


$$Precision=\frac{TP}{TP+FP}$$ (8)

Recall represents the proportion of actual positive samples in the test set that are correctly predicted by the classification model.


$$Recall=\frac{TP}{TP+FN}$$ (9)

The F1-Score is the harmonic mean of precision and recall, offering a balanced metric that accounts for both.


$$F\text{1}=\frac{\text{2}\times Precision\times Recall}{Precision+Recall}$$ (10)

In the formula, TP indicates the number of samples the model correctly predicts as positive, TN denotes the number correctly predicted as negative, FP represents the number incorrectly predicted as positive, and FN refers to the number incorrectly predicted as negative.

Additionally, during model training, the loss value, FLOPs, inference time, and parameter count are also key performance evaluation metrics. A faster decrease in the loss value indicates quicker convergence and a lower value suggests better robustness and performance. FLOPs affect the model’s computational complexity, inference speed, and power consumption. Models with high FLOPs require more computational resources and consume more energy. Reducing FLOPs improves the model’s efficiency in resource-constrained environments, such as mobile devices. Inference time denotes the average processing time required per image during model inference, serving as an indicator of the model’s real-time performance and deployment efficiency. The parameter count determines the model’s storage requirements, training time, and computational resources. Models with more parameters have more substantial expressive capabilities but are more prone to overfitting. Reducing the parameter count can lower resource consumption and enhance deployment efficiency on resource-constrained devices.

### 4.3 Training parameters settings

The parameter settings for the model training process are provided in Table 3.

**Table 3:** Values of experimental parameters.

Parameters	Values
Optimizer types	SGD
Momentum	0.01
Weight_decay	0.9
Learning rate	0.0008
Batch-size	32
Number of epochs	60

## 5 Results and analysis

### 5.1 Comparative tests on different parameters

This study investigates the impact of different batch sizes (4, 8, 16, 32) and learning rates (0.1, 0.01, 0.001) on the model’s disease detection performance. A total of twelve experiments were performed, with the training and testing outcomes for each group summarized in Table 4. The results showed that both batch size and learning rate significantly impacted the model’s performance during training and testing. Furthermore, to ensure the robustness and reliability of the experimental results, this study presents the average values for each batch performance in the Table. This approach aims to minimize the impact of random fluctuations and strengthen the statistical validity of the findings.

**Table 4:** Accuracy and loss values of ES-ShuffleNet model training and prediction.

Number	Batch-size	Learning rate	Train accuracy	Testing accuracy	Train loss	Testing loss
1	4	0.1	98.49%	94.15%	0.0500	0.1935
2		0.01	98.74%	94.15%	0.0435	0.1969
3		0.001	98.71%	95.98%	0.0417	0.1803
4		average	98.65%	94.76%	0.0451	0.1902
5	8	0.1	98.74%	95.25%	0.0439	0.1960
6		0.01	98.83%	95.61%	0.0409	0.1801
7		0.001	98.66%	95.98%	0.0440	0.1461
8		average	98.74%	95.61%	0.0429	0.1741
9	16	0.1	98.92%	95.80%	0.0381	0.1314
10		0.01	98.85%	97.07%	0.0372	0.1319
11		0.001	99.01%	95.98%	0.0353	0.1634
12		average	98.93%	96.28%	0.0369	0.1422
13	32	0.1	98.85%	93.60%	0.0377	0.2449
14		0.01	98.71%	91.41%	0.0437	0.3190
15		0.001	98.76%	94.88%	0.0362	0.1807
16		average	98.77%	93.30%	0.0392	0.2482

#### 5.1.1 Impact of batch-size on model.

**Fig 11:**
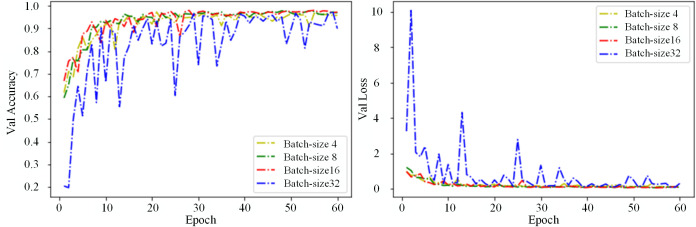
Validation accuracy and loss curve for different Batch-sizes. a: Val accuracy; b: Val loss.

In deep learning, Batch Size denotes the number of training samples processed in each forward and backward pass. The size of the Batch Size significantly impacts the model’s training process. Smaller Batch Sizes result in longer training times and more fluctuation in the curve. In comparison, larger Batch Sizes lead to smoother training. However, they may cause overfitting, especially when the training set has insufficient samples or low data diversity, which can affect the model’s ability to generalize. The experimental results in Fig 11. show that Batch Size influences the convergence speed and stability of model training. When the initial learning rate is 0.01, increasing the batch size from 4 to 32 accelerates the convergence of the accuracy and loss values on the validation set. However, it increases the oscillation, leading to enhanced training fluctuation and reduced generalization ability. When the batch size is 16, the model achieves the best accuracy and convergence stability. Therefore, a batch size of 16 was selected for training the model on the dataset.

#### 5.1.2 Impact of learning rates on the model.

**Fig 12:**
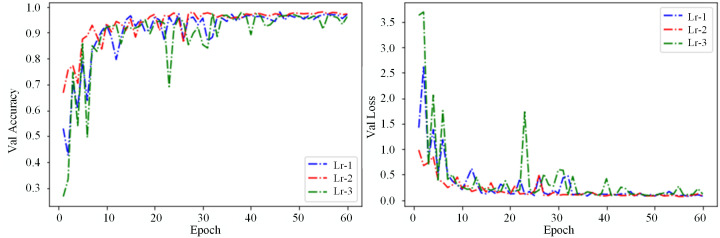
Validation accuracy and loss curves for different Learning Rates. a: Val accuracy; b: Val loss.

The learning rate sets the step size for parameter updates in each iteration, serving as a scaling factor for weight changes during backpropagation. A more minor learning rate results in slower model updates, extended convergence time, and a gradual decrease in the loss function, significantly increasing training duration. Conversely, a tremendous learning rate accelerates convergence but may skip the optimal point or fail to converge, causing instability in the training process and oscillations in the loss function curve. Experiments were carried out to assess how the learning rate affects model performance, with values set at 0.1, 0.01, and 0.001, while keeping all other parameters unchanged. Fig 12. illustrates the variation curves of validation accuracy and loss values under different learning rates when the batch size was set to 16. The results indicate that with a learning rate of 0.1, the loss values oscillated significantly, and the accuracy was low; with a learning rate of 0.001, the model exhibited overfitting; with a learning rate of 0.01, the training achieved the best results, characterized by high accuracy and stable curves.

### 5.2 Corn leaf disease recognition experiment

Table 5 presents the specific evaluation metrics for the ES-ShuffleNetV2 model’s recognition experiments. The model achieved an average precision of 97.07% in disease recognition, demonstrating its capability for rapid and accurate identification of corn diseases, thereby meeting the expected objectives of the model improvement. Despite the potential influence of class imbalance on model performance, the consistently high values of Precision, Recall, and F1-Score observed across all categories collectively indicate that the model was not significantly compromised in this case.

**Table 5:** The evaluation metrics for recognition experiments.

Recognition accuracy	Precision	Recall	F1_Score	Accuracy
Commom rust	0.96	0.98	0.97	0.97
Bipolaris maydis	0.98	0.96	0.97	
Curvularia lunata(wakker)boed spot	1.00	0.98	0.99	
Northern leaf blight	0.96	0.97	0.97	
Own spot	0.89	0.90	0.90	
Sheath blight	0.99	1.00	0.99	
Average	0.97	0.97	0.97	

### 5.3 Comparison of attention mechanism modules

**Fig 13:**
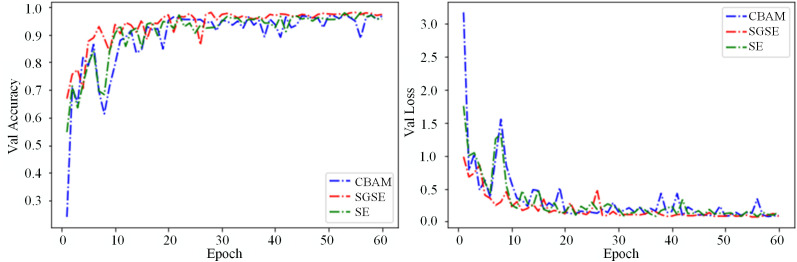
Validation accuracy and loss curve for different attention mechanisms. a: Val accuracy; b: Val loss.

The SGSE module, proposed as an enhancement to the SE module, improves spatial and channel attention for corn disease feature maps. Likewise, the CBAM (Convolutional Block Attention Module) combines attention mechanisms across both channel and spatial dimensions [39]. To evaluate the effectiveness of the SGSE module, comparative experiments were conducted under identical conditions by incorporating various attention modules into the model. As presented in Table 6, the results demonstrate that the enhanced SGSE module surpasses other attention mechanisms in most metrics—such as Precision, Recall, F1-Score, Training Accuracy, and Testing Accuracy—with only slight differences in a few measures. Moreover, the SGSE module achieves this enhanced performance while maintaining comparable model complexity and computational cost relative to SE and CBAM, demonstrating that the improvement in accuracy is not achieved at the expense of significantly increased parameters or FLOPs. Fig 13 illustrates the validation accuracy and loss curves corresponding to various attention mechanisms. In comparison with SE and CBAM, which exhibit significant oscillations in their curves, the SGSE module demonstrates a smoother trend and higher test accuracy, reflecting superior training stability and generalization performance.

**Table 6:** Evaluation metrics of different attention mechanisms.

Attention module	Precision	Recall	F1_Score	Train accuracy	Testing accuracy	FLOPs	Params
CBAM	95.94%	95.80%	95.87%	98.69%	95.80%	7,112,350	884,340
SE	95.25%	95.06%	95.16%	99.02%	95.06%	7,112,548	884,388
SGSE	97.07%	97.07%	97.07%	98.85%	97.07%	7,112,563	884,388

### 5.4 Comparison of the model before and after pruning

Under the same experimental conditions, this study conducted comparative experiments on both the baseline and the improved models before and after pruning, as shown in Table 7. The results demonstrate that, compared to the unpruned model, the pruned ES-ShuffleNetV2 disease recognition model experienced a 0.93% decrease in training accuracy, while its Precision, Recall, and F1-Score each improved by 0.67%,0.73%,0.70%. Regarding computational efficiency, the number of FLOPs decreased from 10,200,613–7,112,563, representing a reduction of 30.27%, and the number of parameters decreased from 1,272,060–884,388, a reduction of 30.48%, respectively, thereby satisfying the lightweight requirements for deployment on resource-constrained devices. Although the baseline model also experienced significant reductions in computational complexity after pruning, it achieved slight improvements in accuracy, which further confirms the effectiveness of the pruning strategy. These findings validate the effectiveness and necessity of applying pruning to the proposed model.

**Table 7:** Comparison of evaluation metrics before and after pruning the model.

	Precision	Recall	F1_Score	Train accuracy	Testing accuracy	FLOPs	Params
Before pruning	96.40%	96.34%	96.37%	99.78%	96.34%	10,200,613	1,272,060
After pruning	97.07%	97.07%	97.07%	98.85%	97.07%	7,112,563	884,388
Before pruning(base)	95.47%	95.43%	95.45%	99.27%	95.43%	10,198,163	1,271,742
After pruning(base)	95.89%	95.80%	95.84%	99.21%	95.80%	7,110,113	884,070

### 5.5 Contrast experiments of different activation functions

To investigate how various activation functions affect model performance, a comparative experiment was conducted utilizing three widely adopted activation functions: Mish, Swish, ReLU, and ELU [40,41]. As presented in Table 8, all activation functions demonstrated robust performance across various metrics. Notably, ELU attained the top values in Precision, Recall, and F1_Score, with each metric reaching 97.07%. Furthermore, ELU exhibited superior inference efficiency, achieving an average inference time of 9.31 ms per image, surpassing both Mish, ReLU and Swish. These findings indicate that ELU is more appropriate as the activation function for this model when considering both accuracy and real-time performance.

**Table 8:** Evaluation metrics of different activation functions.

Activation functions	Precision	Recall	F1_Score	Train accuracy	Testing accuracy	Inference time for processing a single image
Mish	96.56%	96.53%	96.54%	99.22%	96.53%	9.38ms
Swish	96.37%	96.34%	96.36%	99.21%	96.34%	9.34ms
ReLU	96.13%	95.98%	96.06%	99.38%	95.98%	9.56ms
ELU	97.07%	97.07%	97.07%	98.85%	97.07%	9.31ms

### 5.6 Ablation experiment

Ablation experiments were designed to demonstrate the impact of each improvement on the model. The outcomes of these ablation studies are summarized in Table 9. Based on ShuffleNet V2, the network model proposed in this paper is ES-ShuffleNet V2. The network obtained by modifying the activation function of ShuffleNet V2 to ELU is called E-ShuffleNet V2. The network obtained by adding the improved SGSE attention mechanism to the ShuffleNet V2 network is called S-ShuffleNet V2.

**Table 9:** ES-ShuffleNetV2 network ablation experiments.

Network model	ELU	SGSE	Precision	Recall	F1_Score	Accuracy	Test accuracy
E-ShuffleNet V2	✔^a^	✗^b^	95.98%	95.80%	95.89%	95.80%	95.80%
S-ShuffleNet V2	✗	✔	96.13%	95.98%	96.06%	95.98%	95.98%
ES-ShuffleNet V2	✔	✔	97.07%	97.07%	97.07%	97.07%	97.07%
ShuffleNet V2	✗	✗	95.47%	95.43%	95.45%	95.43%	95.43%

^a^means the module was used in the experiment; ^b^ means the module was not used in the experiment.

The comparison between the training results of ShuffleNet V2 and ES-ShuffleNet V2 indicates that the recognition accuracy of the ES-ShuffleNetV2 model improved by 1.64%, while the testing accuracy, recall, F1-Score, and precision each improved by 1.64%, 1.64%, 1.62%, 1.60%. Furthermore, the comparison between E-ShuffleNet V2 and ShuffleNet V2 demonstrates that the ELU activation function offers stronger nonlinear representational capabilities than the ReLU function, resulting in a 0.37% increase in recognition accuracy. In addition, the comparison between S-ShuffleNet V2 and ShuffleNet V2 indicates that the integration of the improved SGSE attention mechanism effectively enhances the model’s feature representation ability, leading to a 0.55% increase in recognition accuracy. Based on these findings, this study adopts the ES-ShuffleNet V2 model for corn disease detection.

### 5.7 Confusion matrix

**Fig 14:**
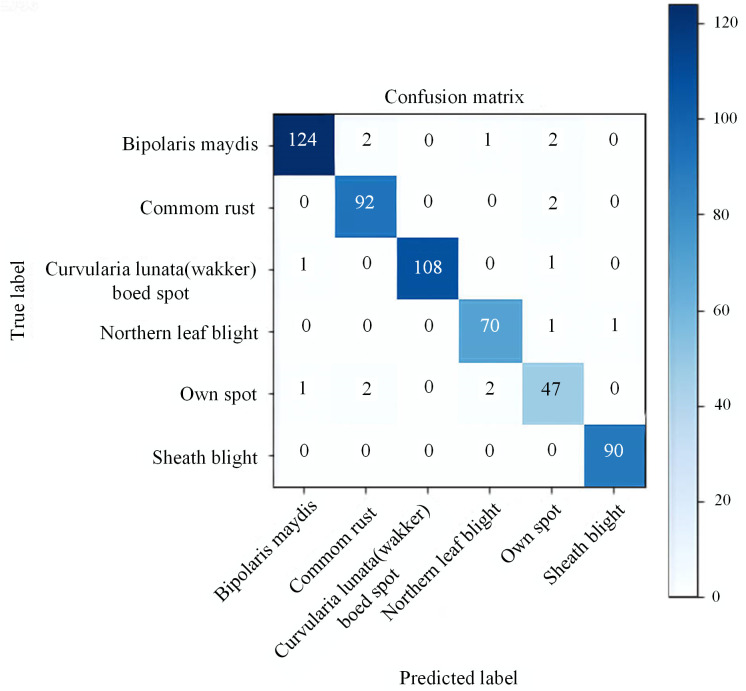
Confusion matrix of the ES-ShuffleNetV2 network model.

Confusion Matrix is a tool for assessing the performance of a classification model, as shown in Fig 14. It is a two-dimensional structure that visually represents the model’s prediction results, allowing for a comprehensive assessment of accuracy, precision, recall, and F1 score. Rows correspond to the true categories, while columns correspond to the categories predicted by the model. From the confusion matrix, it can be observed that the model achieves high accuracy for Common rust, Bipolaris maydis, Curvularia lunata(wakker) boed spot, Sheath blight, Northern leaf blight, with accuracy reaching 100%. Although the accuracy of Own spot is slightly lower due to considerable feature variations among disease samples and complex backgrounds, the overall precision remains nearly 100%.

### 5.8 Cross dataset validation

To further evaluate the generalization capability of the proposed ES-ShuffleNet V2 model under different data distributions, a cross-dataset validation experiment was conducted. The validation dataset was derived from PlantVillage, from which four representative categories were selected, with 500 images per category: a. Cercospora leaf spot, b. Common rust, c. Healthy, and d. Northern leaf blight.

**Fig 15:**
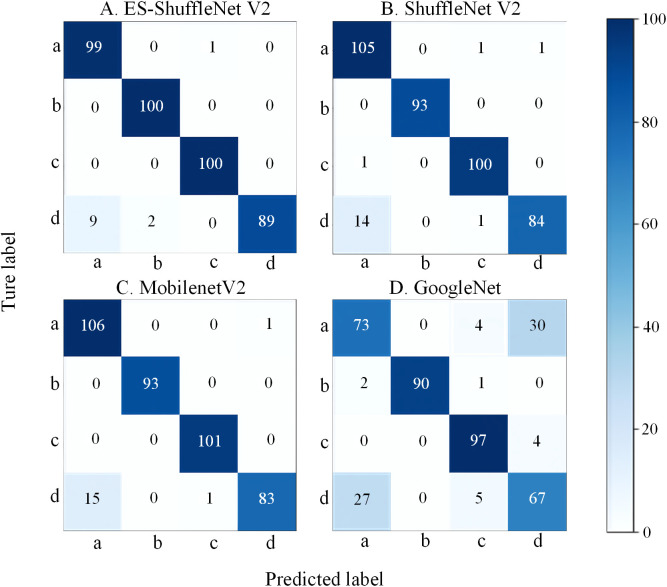
Confusion matrices for cross-dataset validation. a: Cercospora leaf spot; b: Common rust; c: Healthy, d: Northern leaf blight.

The experimental results are shown in Fig 15 and Table 10. The confusion matrices demonstrate that ES-ShuffleNet V2 exhibits clearer diagonal patterns and fewer misclassifications, indicating superior cross-dataset recognition performance. Among all models, ES-ShuffleNet V2 achieved the best results, significantly outperforming MobileNetV2, ShuffleNet V2, and GoogleNet. Further analysis revealed that most misclassifications occurred between Cercospora leaf spot (a) and Northern leaf blight (d), which is consistent with real-world scenarios where these two diseases exhibit highly similar visual symptoms. In contrast, GoogleNet performed the worst, with an Accuracy of only 81.75%, and showed especially high misclassification rates for classes a and d.

**Table 10:** Evaluation metrics of different models in cross-dataset validation.

Network model	Precision	Recall	F1_Score	Accuracy
GoogleNet	81.95%	81.75%	81.85%	81.75%
MobilenetV2	96.32%	95.75%	96.04%	95.75%
ShuffleNet V2	95.97%	95.50%	95.73%	95.50%
ES-ShuffleNet V2	97.28%	97.00%	97.14%	97.00%

Overall, these findings demonstrate that ES-ShuffleNet V2 not only performs well on the self-constructed dataset but also maintains high recognition accuracy when applied to datasets with different distributions, confirming its practicality and strong generalization ability for real-world agricultural applications.

### 5.9 Robustness verification

To ensure that the high recognition accuracy of the proposed model was not the result of overfitting or memorization, a robustness evaluation was performed under three challenging conditions: Gaussian noise with a standard deviation of 10, Gaussian blur with a kernel size of 5 × 5, and illumination variation achieved by increasing brightness and contrast by approximately 20%.

The results are summarized in Table 11. Under standard conditions, the proposed ES-ShuffleNet V2 achieved an average accuracy of 97.07%, exceeding that of the baseline ShuffleNet V2 95.43%. When Gaussian noise was introduced, the accuracy of ES-ShuffleNet V2 decreased slightly to 95.14%, while the baseline dropped to 93.97%, indicating stronger resilience to random perturbations. Under Gaussian blur, ES-ShuffleNet V2 maintained 94.52% accuracy, outperforming the baseline by 1.1%. The most significant performance gap appeared under brightness variation: ES-ShuffleNet V2 achieved 88.48% accuracy compared with 81.17% for the baseline, a 7.31% improvement. These results demonstrate that the proposed model maintains high recognition accuracy even under challenging imaging conditions, showing stronger robustness and generalization ability than the baseline.

**Table 11:** Performance comparison of ES-ShuffleNet V2 and ShuffleNet V2 under different perturbation conditions.

Model	Test type	Accuracy	Precision	Recall	F1_Score
ES-ShuffleNet V2	Original	97.07%	97.07%	97.07%	97.07%
	Gaussian noise	95.14%	95.88%	95.14%	95.51%
	Gaussian blur	94.52%	94.63%	94.52%	94.57%
	Bright	88.48%	88.90%	88.48%	88.69%
ShuffleNet V2	Original	95.43%	95.47%	95.43%	95.45%
	Gaussian noise	93.97%	94.10%	93.97%	94.03%
	Gaussian blur	93.42%	93.43%	93.42%	93.42%
	Bright	81.17%	82.34%	81.17%	81.75%

### 5.10 Model performance comparison experiment

**Fig 16:**
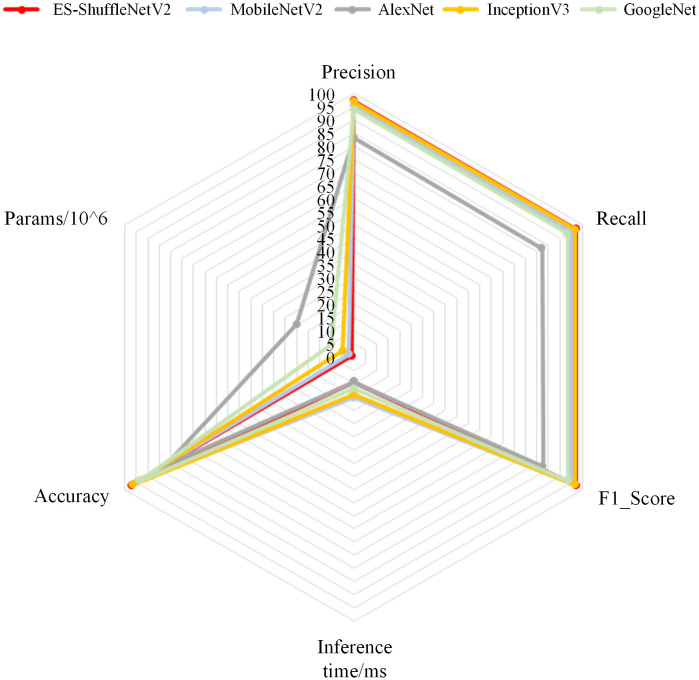
Evaluation metrics for different models.

The proposed ES-ShuffleNet V2 model is compared with the MobilenetV2, GoogleNet, AlexNet, and InceptionV3 models. After 60 iterations, the final evaluation metrics for each model are shown in Fig 16 [42–45]. The ES-ShuffleNet V2 model, improved based on ShuffleNet V2, achieves higher classification accuracy, lower parameter count, and superior performance.

### 5.11 Discussion

The ES-ShuffleNetV2 model proposed in this study demonstrated strong performance in corn leaf disease recognition, achieving an average recognition accuracy of 97.07%, thus enabling accurate identification of corn diseases. By incorporating the SGSE module to enhance the model’s feature representation capability, the model outperformed other attention mechanisms in terms of Precision, Recall, and F1-Score, confirming the effectiveness of the proposed module. However, the current scope of disease categories is limited to six common corn diseases, which may restrict the model’s applicability in more complex and diverse agricultural scenarios. Comparative experiments on activation functions revealed that the ELU function provided superior nonlinear representational capability compared to ReLU and other commonly used functions. This improvement not only enhanced recognition accuracy but also accelerated detection speed, confirming the advantage of ELU in this task. Furthermore, a pruning strategy was applied to reduce the model’s computational complexity and parameter count; as a result, FLOPs and parameters of ES-ShuffleNetV2 were reduced by 30.26% and 30.45%, respectively, compared to the baseline model, achieving both model simplification and performance enhancement. These results highlight the effectiveness of pruning in lightweight model design.

Moreover, to eliminate potential data leakage, the dataset was re-divided at the raw image level, and data augmentation was applied only to the training set. After this correction, the overall accuracy slightly decreased, indicating that the new test set provides a stricter and more realistic evaluation. Nevertheless, the model maintained strong generalization performance under this more rigorous setup. Importantly, cross-dataset validation demonstrated that ES-ShuffleNetV2 maintains high recognition accuracy across datasets with different distributions. This finding indicates that the model possesses strong generalization capability, further supporting its practical applicability in real-world agricultural scenarios. Although the accuracy of both models decreased under noisy, blurred, and uneven lighting conditions, this degradation is reasonable because such perturbations distort key visual details and reduce image clarity. Even so, the smaller drop observed in ES-ShuffleNetV2 suggests that the SGSE module improves the network’s ability to capture meaningful spatial information, while the ELU activation function helps maintain more stable responses when brightness changes. These results indicate that the proposed improvements not only enhance accuracy under normal conditions but also provide better robustness to real-world imaging variations.

In addition, batch size and learning rate were found to have a significant impact on the model’s convergence speed and generalization performance. Based on accuracy and convergence stability, a batch size of 16 and a learning rate of 0.01 were selected as the optimal training settings. While the recognition accuracy for certain diseases approached 100%, the accuracy for Own Spot disease was slightly lower due to variations in sample characteristics and complex image backgrounds.

Future work will focus on several aspects to further enhance the applicability and impact of the ES-ShuffleNetV2 model. One direction involves extending disease recognition to additional crop species and verifying the model’s generalization through cross-regional and cross-seasonal datasets. Another focus will be on incorporating multi-modal information—such as hyperspectral data and environmental factors—to improve the system’s robustness under diverse field conditions. Moreover, lightweight optimization techniques, including model quantization and knowledge distillation, will be investigated to support deployment on mobile or embedded devices for real-time disease monitoring. Finally, model interpretability will be further explored using Grad-CAM and related visualization methods, so that the model’s decision process becomes more transparent and useful for agricultural practitioners. These efforts together are expected to extend the current study and increase its practical value in precision agriculture.

## 6 Conclusion

This paper proposes an improved corn leaf disease recognition model, ES-ShuffleNetV2, based on ShuffleNetV2, intended to overcome the limitations of existing models in resource-limited settings, including high computational demands and constrained recognition accuracy. By introducing the improved SGSE attention module and replacing the activation function with ELU, the model’s feature extraction capability and training efficiency were significantly enhanced. In addition, a pruning strategy was employed to reduce model complexity, achieving accurate disease recognition with lower computational resource requirements. The ES-ShuffleNetV2 model was validated through corn leaf disease recognition experiments and achieved an average accuracy of 97.00%. It maintained high recognition accuracy across all six corn disease categories. Compared with other attention mechanisms, the SGSE module delivered the best performance in terms of accuracy. Comparative experiments on activation functions demonstrated that the ELU function achieved both the highest accuracy and the shortest inference time, with a single-image inference time of only 9.31ms. Ablation experiments confirmed the effectiveness of each structural improvement, among which the SGSE module had the most notable impact. The ES-ShuffleNetV2 model achieved a test accuracy of 97.07% in the ablation study, representing a 1.64% improvement over the baseline. It should be emphasized that cross-dataset and robustness evaluations demonstrate that ES-ShuffleNetV2 maintains high recognition accuracy across different data distributions, underscoring its strong generalization ability and practical applicability in real-world agricultural disease monitoring. Future research will aim to apply the model to a broader spectrum of crop diseases and further optimizing feature extraction strategies to enhance robustness and scalability.
